# Influence of herd immunity on norovirus: a long-term field study of repeated viral gastroenteritis outbreaks at the same facilities

**DOI:** 10.1186/s12879-023-08251-7

**Published:** 2023-04-26

**Authors:** Makoto Kumazaki, Shuzo Usuku

**Affiliations:** Microbiological Testing and Research Division, Yokohama City Institute of Public Health, 2-7-1 Tomiokahigashi, Kanazawa-Ku, Yokohama, Kanagawa 236-0051 Japan

**Keywords:** Norovirus, Sapovirus, Rotavirus, Acute gastroenteritis, Outbreak, Genotype, Epidemiology, Herd immunity

## Abstract

**Background:**

Viral acute gastroenteritis (AG) is detected worldwide annually. Outbreaks caused by viruses associated with gastroenteritis have been reported repeatedly at the same facilities in Yokohama, Japan over several years. We investigated the statuses of these repeated outbreaks to consider herd immunity at the facility level.

**Methods:**

Between September 2007 and August 2017, 1459 AG outbreaks were reported at 1099 facilities. Stool samples were collected for virological testing, and the norovirus gene was amplified and sequenced to determine the genotype using the N-terminal region of the capsid.

**Results:**

The outbreaks were caused by norovirus, sapovirus, rotavirus A, and rotavirus C. Norovirus was consistently predominant over the 10-year period. Of 1099 facilities, 227 reported multiple outbreaks, of which norovirus-only combinations accounted for 76.2%. More outbreaks were due to different genotype combinations than the same genotype combinations. For facilities that experienced two norovirus outbreaks, the average interval between outbreaks was longer for groups with the same combinations than for groups with different genogroup or genotype combinations, although no statistically significant differences were observed. At 44 facilities, outbreaks occurred repeatedly during the same AG season, and most exhibited combinations of different norovirus genotypes or viruses. Among 49 combinations with the same norovirus genotype at the same facilities over 10 years, the most prevalent genotypes were combinations of genogroup II genotype 4 (GII.4), followed by GII.2, GII.6, GII.3, GII.14, and GI.3. The mean interval between outbreaks was 31.2 ± 26.8 months for all combinations, and the mean intervals were longer for non-GII.4 genotype cases than for GII.4 cases, and statistically significant differences were observed (t-test, *P* < 0.05). Additionally, these average intervals were longer for kindergarten/nursery schools and primary schools than for nursing homes for older adults (t-test, *P* < 0.05).

**Conclusions:**

Repeated AG outbreaks at the same facilities in Yokohama during the 10-year study period included mainly norovirus combinations. Herd immunity at the facility level was maintained for at least the same AG season. Norovirus genotype-specific herd immunity was maintained for an average of 31.2 months during the study period, and these intervals differed depending on genotype.

**Supplementary Information:**

The online version contains supplementary material available at 10.1186/s12879-023-08251-7.

## Background

Acute gastroenteritis (AG) outbreaks occur worldwide annually and can be caused by viruses, bacteria, and parasites. Norovirus and rotavirus are representative viruses causing infectious gastroenteritis outbreaks, and these diseases can affect everyone [1]. Sapovirus also causes viral gastroenteritis, and several sapovirus outbreaks have been reported [2]. In Yokohama, Japan, norovirus, rotavirus, and sapovirus outbreaks have occurred in various settings annually [3–5], and outbreaks due to these viruses have been repeatedly reported at the same facilities in Yokohama over several years.

Based on the major capsid (VP1) gene, norovirus strains can be classified into 10 genogroups: GI–GX [1, 6–8]. Most noroviruses detected in human infections belong to genogroups GI and GII, which contain nine and 26 genotypes, respectively [6–8]. Norovirus strains can also be divided into polymerase genogroups and genotypes using a partial region of the RNA-dependent RNA polymerase (RdRp) gene [6–8]. A recent mathematical model based on community transmission estimated that immunity to norovirus likely lasts 4–8 years [9]. Human challenge studies have shown poor cross-reactivity between GI and GII viruses and have reported that most repeat infections were due to genotypes that differed from those of previous infections [10, 11]. Genotype-specific herd immunity may influence norovirus outbreaks at the facility level [12]. Additionally, sapovirus is antigenically diverse and is classified into multiple genogroups and genotypes [2]. A case of reinfection with sapoviruses from different genogroups was recently reported, although protective immunity among humans to sapovirus infections remains unknown [13, 14]. Moreover, rotaviruses belong to species A, B and C (RVA, RVB, and RVC, respectively) infect humans and other animals. Epidemiologically, RVA is the most important for human infection and disease and has been further classified using various approaches [15]. A previous study reported that natural rotavirus infection in infants confers protection against subsequent infections, and this protection increases with each new infection and reduces diarrheal severity [16].

The presence and proximity of immune individuals reduces the risk of infection among susceptible individuals in a population [17], which consequently results in fewer outbreaks. However, to date, facility-level herd immunity is rarely discussed. When considering herd immunity at the facility level, the influence of human populations with different immune histories, such as transition of people in facilities over time and students in different grades, cannot be avoided. However, such factors cannot be fully captured actually in every facility. In addition, it is difficult to obtain samples continuously from patients in the facilities over the long term, although it may be better to use seroepidemiology to evaluate the herd immunity for the viruses that mutate frequently and has many genotypes. Due to these limitations, it may be difficult to evaluate the herd immunity at the facility level accurately. However, we believed that we might be able to identify some trends by including more facilities in our analysis. In this study, we performed epidemiological and genetic analyses of repeated AG virus-associated outbreaks at the same facilities in Yokohama, Japan over a period of 10 years and considered herd immunity at the facility level.

## Materials and methods

### Sample collection and outbreak definition

AG outbreaks in Japan are reported to local government public health centers by order of the Ministry of Health, Labour and Welfare. These health centers then conduct field investigations. The main symptoms of AG include vomiting and watery diarrhea, and other symptoms may include fever, abdominal cramps, nausea, muscle aches, and headache. Virological test was performed if a person’s main symptom was vomiting or watery diarrhea and viral infection was suspected based on incubation period. Between September 2007 and August 2017, 1459 AG outbreaks, suspected to be either foodborne or due to person-to-person transmission, were reported, and a total of 9900 stool samples were collected for virological testing by the Health and Social Welfare Bureau, Yokohama, Japan, along with epidemiological information from each outbreak. Outbreaks were defined as the AG having occurred in a setting where (1) 10 or more people or more than half of those present were infected, or (2) the number of cases exceeded normal trends. An AG season was defined as the 12-month period from September through August of each year. Outbreaks were considered terminated when no new incidents occurred within the facility for 3 days from the last onset according to Health and Social Welfare Bureau, Yokohama criteria. The pathogen of AG outbreak was confirmed if more than one or two AG cases tested positive for either viruses by real-time RT-PCR.

### Detection of viruses using real-time RT-PCR

A 10% stool suspension was prepared by mixing each stool sample with 1 × phosphate-buffered saline (pH 7.4), followed by centrifugation at 10,000 × *g* for 10 min at 4 °C. Viral RNA was extracted from the supernatants using the RNeasy Mini Kit (Qiagen, Hilden, Germany) per the manufacturer’s instructions. Real-time RT-PCR detection of norovirus, sapovirus, RVA, and/or RVC was performed using a SmartCycler II (Cepheid, Sunnyvale, CA, USA) with a QuantiTect Probe RT-PCR Kit (Qiagen). The primers and probes used to detect these viruses have been previously described [18–22].

### RT-PCR for norovirus genotyping

One positive specimen was selected randomly from each norovirus outbreak. In some cases that showed two types of norovirus (GI and GII) by real-time PCR, two or more specimens in each outbreak were selected. Of 5467 norovirus positive samples, 1417 stool samples were subjected to gene amplification of the N-terminal shell region to determine the genotype. RT-PCR was performed using the TaKaRa One Step RNA PCR Kit (Takara Bio Inc., Shiga, Japan). The PCR primers used were previously described [23, 24].

### PCR for the partial RdRp and VP1 regions of norovirus strains

Some strains were analyzed for the partial RdRp and VP1 regions of norovirus. cDNA was synthesized from the extracted viral RNA with SuperScript III Reverse Transcriptase (Invitrogen, Carlsbad, CA, USA), and random hexamer primers (Takara Bio Inc., Shiga, Japan) were used as the PCR template. PCR was performed for amplification with TaKaRa Ex Taq DNA polymerase (Takara Bio Inc., Shiga, Japan). Additional file 1 lists the primers used for PCR. Our designed PCR primers were used under the following cycling conditions: 95 °C for 1 min, followed by 40 cycles of 95 °C for 15 s, 55 °C for 30 s, and 72 °C for 1 min. The other primers were used as described previously [23–32].

### Data analysis

The nucleotide sequences of the purified PCR products (QIAquick PCR Purification Kit, Qiagen) were determined using the BigDye Terminator Cycle Sequencing Kit (Applied Biosystems, Foster City, CA, USA) and a Genetic Analyzer 3130 or 3500 (Applied Biosystems) per the manufacturer’s instructions. The obtained data were used to determine the norovirus genotype using the web-based Norovirus Genotyping Tool, Version 2.0 software [33]. The obtained partial RdRp and VP1 region data were used to construct a phylogenetic tree on the basis of nucleotide sequences with the neighbor-joining method using MEGA 6 software (http://www.megasoftware.net/) with 1,000 bootstrap replicates. Genogroup/Genotype classification of norovirus were determined based on the latest 2019 reference [8]. The sequences reported herein were deposited in the DDBJ/GenBank/EMBL databases under accession numbers LC720153–LC720176.

### Statistical analysis

Statistical analysis was performed using IBM SPSS Statistics Version 27 (IBM, Armonk, NY, USA). Student’s t-test or Welch’s t-test was used to determine significant differences between group means. *P* values < 0.05 were considered statistically significant.

## Results

### Outbreaks due to viral AG

**Fig. 1:**
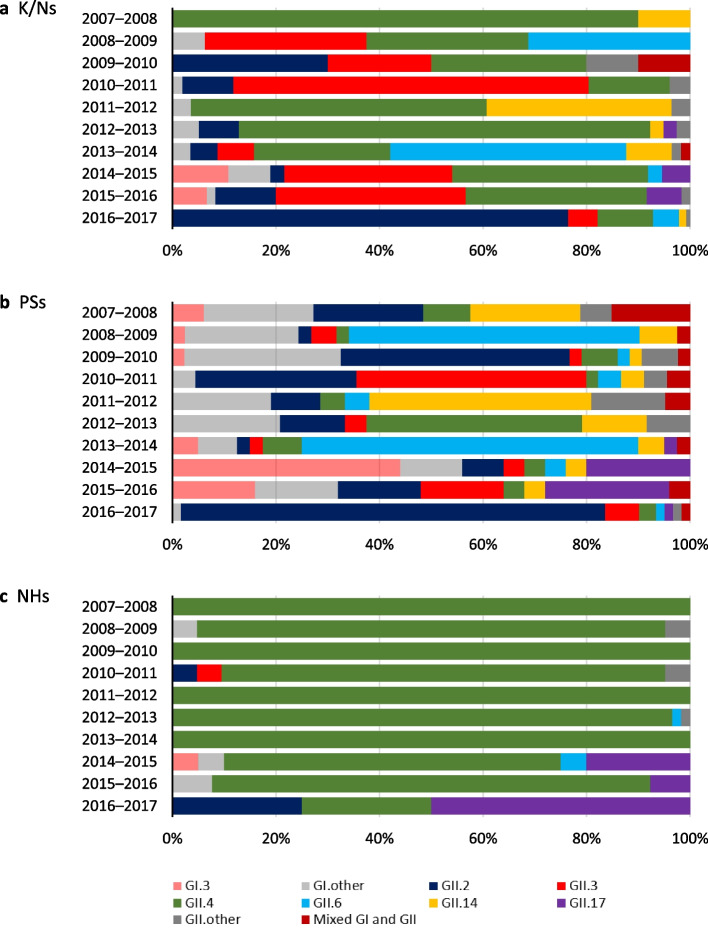
Distribution of norovirus genotypes in **a** K/Ns, **b** PSs, and **c** NHs by season. Distributions from 2007–2008 to 2014–2015 season were confirmed in our previous study (ref. 3). GII.other includes undetermined genotypes for GII. K/Ns: Kindergarten/nursery schools; PSs: Primary schools; NHs: Nursing homes for the aged

During 10 consecutive 12-month periods starting in September 2007, 1459 AG outbreaks were reported in Yokohama. Table 1 summarizes the outbreaks by season. Almost AG outbreaks were due to person-to-person cases, although included some food poisoning cases. Outbreaks due to norovirus, sapovirus, RVA, or RVC were determined, and norovirus was dominant every season and consistently accounted for 87.4%–96.8% of the total outbreaks. These outbreaks occurred in various settings annually, but mainly in kindergarten/nursery schools (K/Ns), primary schools (PSs), and nursing homes for older adults (NHs). The distribution of settings in which the AG outbreaks occurred differed by causative virus (Additional file 2). Norovirus outbreaks occurred among all patient ages. RVA outbreaks occurred mainly in infants, and 57% occurred in K/Ns. Sapovirus outbreaks occurred mainly in children, and 53% occurred in PSs. All RVC outbreaks occurred in PSs. Additionally, we investigated the norovirus genotype distributions in K/Ns, PSs, and NHs by AG season (Fig. 1) and found that the dominant genotype in each season was altered in PSs.

**Table 1:** Number of virus-associated outbreaks due to acute gastroenteritis in Yokohama, Japan

			Season									
	Total		2007–2008	2008–2009	2009–2010	2010–2011	2011–2012	2012–2013	2013–2014	2014–2015	2015–2016	2016–2017
Total of AG outbreaks	1459	(100%)	114	103	93	171	122	161	162	139	135	259
Virus												
Norovirus^a^	1327	(90.9%)	104	93	90	155	110	150	150	123	118	234
RVA	73	(5.0%)	5	6	1	7	4	3	9	8	10	20
Sapovirus	49	(3.4%)	1	3	2	8	6	8	3	8	6	4
RVC	10	(0.7%)	4	1	0	1	2	0	0	0	1	1
Setting												
K/Ns^b^	504	(34.6%)	11	18	11	59	31	43	67	46	67	151
PSs^b^	413	(28.3%)	40	46	45	54	27	27	40	30	32	72
NHs^b^	256	(17.6%)	30	24	17	21	36	60	25	22	14	7
Restaurants	143	(9.8%)	16	5	10	19	14	16	13	19	14	17
Welfare facilities	42	(2.9%)	1	4	5	3	6	4	7	8	1	3
Hospitals	31	(2.1%)	10	1	2	5	4	4	3	2	0	0
Junior and senior high schools, universities	16	(1.0%)	5	1	3	1	0	1	0	2	0	3
Others^c^	54	(3.7%)	1	4	0	9	4	6	7	10	7	6

^a^Number of norovirus outbreaks from 2007–2008 to 2013–2014 were confirmed in our previous study (ref. 26)

^b^K/Ns: Kindergarten/nursery schools; PSs: Primary schools; NHs: Nursing homes for the aged

^c^Other settings include the following: home (*n* = 15), trip (*n* = 7), banquet room (*n* = 5), child consultation center (*n* = 4), foster home (*n* = 2), office (*n* = 2), dormitory (*n* = 2), and other unspecified (*n* = 17)

### Repeated AG outbreaks reported at the same facilities

**Fig. 2:**
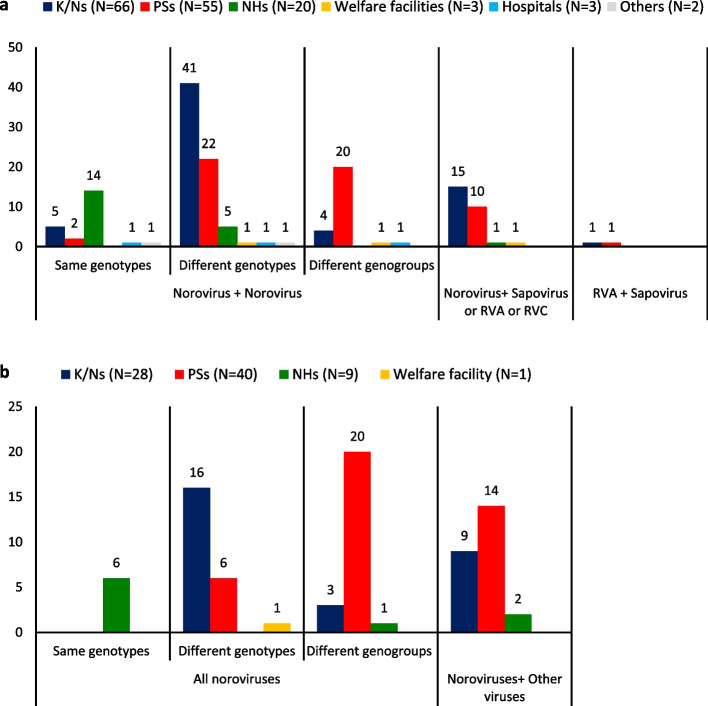
Combinations of AG viruses or norovirus genotypes detected in each setting. **a** AG outbreaks occurred twice. **b** AG outbreaks occurred more than three times. Other settings are one child consultation center and one foster home. Other viruses are sapovirus, RVA, and/or RVC. K/Ns: Kindergarten/nursery schools; PSs: Primary schools; NHs: Nursing homes for the aged

During the study period, 1099 facilities reported AG outbreaks. Of these facilities, 227 reported multiple outbreaks at the same facility, accounting for approximately 20% of all facilities. Table 2 summarizes the repeated AG outbreaks for each setting. Repeated AG outbreaks occurred in 94 K/Ns, 95 PSs, 29 NHs, 4 welfare facilities, 3 hospitals, and 2 other settings, with higher proportions in K/Ns (94/353) and PSs (95/255). The most frequent occurrences for each setting were 10 outbreaks at a K/N (KNo5-7), 7 at a PS (PSo5-3), 5 at an NH (NHo5-1), 3 at a welfare facility (WF3-1), 2 in a hospital (HP2-1–HP2-3), and 2 in other settings (OT2-1 and OT2-2; Additional file 3). No reports of multiple outbreaks at the same facility were confirmed in restaurants, junior or senior high schools, or universities. We investigated the viruses and norovirus genotypes detected in these 227 facilities (Additional file 3), and 76.2% (173/227) had only norovirus outbreaks. Figure 2 summarizes the detected virus combinations.

**Table 2:** Number of facilities where repeated AG outbreaks were reported during 10 AG seasons

	K/Ns^a^		PSs^a^		NHs^a^		Welfare facilities		Hospitals		Restaurants	Junior and senior high schools, universities	Others	
Total outbreaks	504		413		256		42		31		143	16	54	
Facilities	353		255		215		37		28		143	16	52	
Frequency														
1	259		160		186		33		25		143	16	50	
2	66	(KN2-1–KN2-66)	55	(PS2-1–PS2-55)	20	(NH2-1–NH2-20)	3	(WF2-1–WF2-3)	3	(HP2-1–HP2-3)	0	0	2^b^	(OT2-1, OT2-2)
3	16	(KN3-1–KN3-16)	24	(PS3-1–PS3-24)	7	(NH3-1–NH3-7)	1	(WF3-1)	0		0	0	0	
4	5	(KN4-1–KN4-5)	13	(PS4-1–PS4-13)	1	(NH4-1)	0		0		0	0	0	
≥ 5	7	(KNo5-1–KNo5-7)	3	(PSo5-1–PSo5-3)	1	(NHo5-1)	0		0		0	0	0	

() indicates facility name listed in Additional file 3

^a^K/Ns: Kindergarten/nursery schools; PSs: Primary schools; NHs: Nursing homes for the aged

^b^1 child consultation center and 1 orphanage

Figure 2a and Table 3 describe the 149 facilities where two outbreaks occurred at the same facility. Norovirus + norovirus combinations (“norovirus + norovirus”) occurred in 120 facilities, and different genogroup or genotype combinations occurred more frequently than the same combinations. Twenty-three facilities had the same genotype combinations. Of these, 11 NHs, 2 K/Ns, and 1 hospital had different genogroup II genotype 4 (GII.4) variant combinations. Seventy-one facilities had different norovirus GII genotype combinations; these combinations were the most common among the 120 facilities that had “norovirus + norovirus”. K/Ns had different norovirus GII genotype combinations more frequently than other settings (Fig. 2a). Fifteen GII genotype combinations were detected, and GII.2 + GII.4 and GII.3 + GII.4 were the most frequent (Table 3). Twenty-six facilities had different genogroup combinations (GI + GII), and PSs had different genogroup combinations more frequently than other settings (Fig. 2a). Other than “norovirus + norovirus”, 15 facilities had norovirus + sapovirus combinations, 11 had norovirus + RVA combinations, 1 had a norovirus + RVC combination, and 2 had sapovirus + RVA combinations.

**Table 3:** Interval between first and second outbreak for the facilities with two repeated AG outbreaks

Combination type	Number of facility	Number of facility (Number of setting^a^)	Interval between outbreaks (Month)		
			Average	Maximum	Minimum
Total	149		32.3	107	0
Same virus (Norovirus + Norovirus)	120		32.8	107	0
Same genotype	23		36.1	83	0
GII.2	2	(2 K/Ns)	41.5	83	0
GII.3	1	(1 PS)	22	–	–
GII.4	19	(3 K/Ns, 14 NHs, 1 hospital, 1 other)	34.9	83	0
GII.6	1	(1 PS)	61	–	–
Different genogroup or genotype	97		32.0	107	2
Different genotype	71		31.3	107	2
GII.2 + GII.3	8	(5 K/Ns, 3 PSs)	24.1	72	2
GII.2 + GII.4	14	(9 K/Ns, 4 NHs, 1 warefare)	32.9	71	10
GII.2 + GII.6	4	(2 K/Ns, 2 PSs)	34.0	35	32
GII.2 + GII.14	5	(1 K/N, 4 PSs)	29.0	53	13
GII.2 + GII.17	2	(1 K/N, 1 PS)	19.5	30	9
GII.3 + GII.4	13	(12 K/Ns, 1 NH)	36.0	66	13
GII.3 + GII.5	1	(1 K/N)	24	–	–
GII.3 + GII.6	2	(1 K/N, 1 PS)	30.0	35	25
GII.3 + GII.14	3	(2 K/Ns, 1 PS)	22.0	30	13
GII.3 + GII.N.D.^b^	1	(1 K/N)	54	–	–
GII.4 + GII.6	6	(5 K/Ns, 1 NH)	21.0	23	4
GII.4 + GII.14	4	(1 K/N, 3 PSs)	29.5	95	2
GII.4 + GII.17	5	(3 NHs, 1 hospital, 1 other)	48.8	107	28
GII.6 + GII.14	2	(2 PSs)	35.5	53	18
GII.6 + GII.17	1	(1 PS)	14	–	–
Different genogroup (GI + GII)	26		33.9	93	2
Different virus	29		30.2	72	0
Norovirus + Other virus (Sapovirus or RVA or RVC)	27		31.1	72	0
RVA + Sapovirus	2		18.5	33	4

^a^K/Ns: Kindergarten/nursery schools; PSs: Primary schools; NHs: Nursing homes for the aged. Other settings are one child consultation center and one foster home

^b^Undetermined genotype

For these 149 facilities, we investigated the combination types and intervals between the first and second outbreaks (Table 3). The mean interval between the first and second outbreaks was 32.3 ± 22.5 months for all combinations, and the longest interval was 107 months for GII.4 + GII.17. For “norovirus + norovirus”, the mean interval was longer for the same combinations (36.1 ± 22.8 months) than for different genogroup or genotype combinations (32.0 ± 22.4 months). The mean interval was longer for norovirus combinations (32.8 ± 22.5 months) than for other virus combinations (30.2 ± 22.1 months). However, neither were significantly different between groups (*P* > 0.05).

Of 78 facilities that each had more than three outbreaks, all reported at least one norovirus outbreak (Fig. 2b). Furthermore, of these facilities, 53 reported only norovirus outbreaks. Six had the same norovirus genotype, GII.4, and all six were NHs (NH3-1–NH3-4, NH4-1, and NHo5-1). At NH3-2, three outbreaks were all the same variant type (GII.4 Sydney 2012; Additional file 3). Twenty-three facilities had different GII genotypes, and 24 had both GI and GII. Of these 47 facilities, 28 included the same genotypes. The remaining 25 facilities reported noroviruses and other AG virus combinations. Of these, eight included the same norovirus genotypes. Two facilities (KNo5-6, KNo5-7) had two RVA outbreaks, and two (PS4-9, PS4-12) had two sapovirus outbreaks. These outbreaks occurred with different RVA or sapovirus genotypes (data not shown).

### Repeated AG outbreaks during the same season at the same facilities

Forty-four facilities experienced repeated AG outbreaks during the same season: 19 K/Ns, 20 PSs, 4 NHs, and 1 welfare facility. Table 4 summarizes these outbreaks. Four facilities (KNo5-4, KNo5-6, KNo5-7, and PS4-2) experienced repeated AG outbreaks in two seasons, and four facilities (KNo5-5, KNo5-7, PS4-13, and PSo5-3) had three AG outbreaks in the same season. Most of these 44 facilities had combinations of different norovirus genotypes or different viruses. Thirteen facilities had norovirus + RVA and/or sapovirus combinations, 12 had different GII genotype combinations, 10 had different norovirus genogroup combinations, and 1 had different GI genotype combinations. Seven facilities had combinations of the same norovirus genotypes (GI.3, GII.2, GII.3, GII.4, or GII.6), and PSs had more than other settings. PS4-2 had the same genotype combinations of GII.6 in the 2013–2014 season and GI.3 in the 2014–2015 season. Overall, the norovirus genotypes that predominated during each season at each setting were mostly related to repeated outbreaks during the same season (Table 4, Fig. 1). PS4-9 had sapovirus + sapovirus, and these genotypes differed.

**Table 4:** Repeated AG outbreaks during the same season

Setting^a^	AG Season	Dominant norovirus genotype^b^	Facility name	AG virus or norovirus genotype (variant) caused the outbreak		
				1st	2nd	3rd
K/Ns	2008–2009	GII.3, GII.4, GII.6	KN2-49	GII.3	GI.4	
	2009–2010	GII.2, GII.4, GII.3	KN4-5	RVA	GII.3	
	2010–2011	GII.3	KN4-3	GII.3	GII.4 (Den Haag 2006b)	
			KNo5-2	GII.4 (Den Haag 2006b)	GII.3	
			KN2-62	GII.3	Sapovirus	
	2011–2012	GII.4, GII.14	KNo5-6	GI.6	RVA	
	2013–2014	GII.6, GII.4	KNo5-4	GII.4 (Sydney 2012)	GI.7	
			KNo5-7	GII.3	RVA	GII.6
			KN2-58	RVA	GII.6	
			KN2-18	GII.6	GII.4 (Sydney 2012)	
	2014–2015	GII.4, GII.3	KN4-4	GII.4 (Sydney 2012)	GII.3	
			KNo5-4	GII.4 (Sydney 2012)	GI.2	
			KNo5-5	GII.17	RVA	Sapovirus
	2015–2016	GII.3, GII.4	KN2-48	GII.4 (Sydney 2012)	GI.6	
			KN3-10	GI.3	GII.4 (Sydney 2012)	
			KNo5-5	GII.3	GI.6	
			KNo5-7	GII.2	Sapovirus	
	2016–2017	GII.2	KN3-3	GII.3	GII.2	
			KN3-2	GII.2	GII.4 (Sydney 2012)	
			KNo5-6	GII.2	RVA	
			KN2-2	GII.2	GII.2	
			KN3-16	GII.2	GII.4 (Sydney 2012)	
PSs	2007–2008	GII.2, GII.14	PS2-4	GII.4 (Den Haag 2006b)	GII.14	
			PS2-28	GI.3	GII.2	
	2009–2010	GII.2	PS3-7	GII.2	GII.6	
	2010–2011	GII.3, GII.2	PS4-7	GII.3	GII.3	
			PS3-24	Sapovirus	GII.2	
			PS2-3	GII.2	GII.3	
			PS4-13	Sapovirus	GII.3	RVA
			PSo5-1	GII.2	GII.12	
	2011–2012	GII.14	PS4-9	Sapovirus	Sapovirus	
			PSo5-3	GII.12	GI.6	GII.14
	2012–2013	GII.4	PS3-11	GI.6	GII.7	
	2013–2014	GII.6	PS4-2	GII.6	GII.6	
			PS4-8	GII.6	GI.6	
			PS4-4	GI.3	GII.6	
	2014–2015	GI.3, GII.17	PS4-6	GI.2	GI.3	
			PS4-2	GI.3	GI.3	
	2015–2016	GII.17	PS3-5	GI.3	GI.3	
	2016–2017	GII.2	PS3-1	GII.2	GII.2	
			PS3-18	GII.2	Sapovirus	
			PS3-22	GI.4	RVA	
			PS4-3	GII.6	GII.2	
NHs	2011–2012	GII.4	NH2-20	GII.4 (New Orleans 2009)	RVA	
			NHo5-1	GII.4 (Den Haag 2006b)	GII.4 (Den Haag 2006b)	
	2012–2013	GII.4	NH2-3	GII.4 (Sydney 2012)	GII.4 (Sydney 2012)	
			NH3-7	GII.4 (Sydney 2012)	Sapovirus	
Welfare facility	2012–2013	–^c^	WF2-3	GII.4 (Sydney 2012)	Sapovirus	

^a^K/Ns: Kindergarten/nursery schools; PSs: Primary schools; NHs: Nursing homes for the aged

^b^A genotype was defined as dominant if it was associated with over 20% of outbreaks in the corresponding season in each setting (Fig. 1)

^c^Dominant genotype was not determined because only 4 AG outbreaks had occurred in welfare facility in this season

### Repeated norovirus outbreaks due to the same genotype at the same facilities

During the 10-year period, 44 facilities (15 K/Ns, 19 PSs, 9 NHs, and 1 hospital) had norovirus outbreaks of the same genotype (or the same variant type for GII.4; Additional file 4). KNo5-7 (GII.2, GII.6) and PS4-2 (GI.3, GII.6) each experienced two outbreaks of the same genotype combinations. NHo5-1, KNo5-1, and NH3-2 each had three outbreaks of the same genotype (GII.4 Den Haag 2006b, GII.4 Sydney 2012, and GII.4 Sydney 2012, respectively). These outbreaks were treated as two combinations of the 1st + 2nd outbreaks and the 2nd + 3rd outbreaks. Therefore, 44 facilities experienced 49 combinations of the same genotype.

Table 5 summarizes the average interval between these outbreaks by genotype and by setting. The most prevalent genotype was GII.4, followed by GII.2, GII.6, GII.3, GII.14, and GI.3; these genotypes have been dominant in Yokohama (Fig. 1). The mean interval between outbreaks per combination was 31.2 ± 26.8 months for all outbreaks. By genotype, the mean intervals were 39.9 ± 30.4 months for non-GII.4 genotype outbreaks and 18.6 ± 12.1 months for GII.4 outbreaks, and a statistically significant difference was observed (*P* < 0.05). The longest average interval was 47.2 months for GII.3, followed by GII.2, GII.6, GII.14, GII.4, and GI.3. Among GII.4 variants, the average intervals were 23.6 months for GII.4 Den Haag 2006b and 16.9 months for GII.4 Sydney 2012. Thus, the average intervals differed by genotype. By setting, the mean intervals were longer for K/Ns and PSs (36.4 ± 26.2 months and 35.7 ± 30.3 months, respectively) than for NHs (16.8 ± 12.1 months), and a statistically significant difference was observed (*P* < 0.05).

**Table 5:** Average interval between outbreaks by the same genotype at the same facility

	Number of combination	Interval between outbreaks (Month)		
		Average	Maximum	Minimum
Total	49	31.2	90	0
Genotype				
GI.3	2	4.0	7	1
GII.2	13	43.2	90	0
GII.3	5	47.2	75	1
GII.4	20	18.6	47	0
GII.6	6	41.2	67	2
GII.14	3	35.0	51	6
Setting^a^				
K/Ns	17	36.4	83	0
PSs	20	35.7	90	0
NHs	11	16.8	40	0
Hospital	1	13	–	–

^a^K/Ns: Kindergarten/nursery schools; PSs: Primary schools; NHs: Nursing homes for the aged

To clarify the relationship between the strains in each combination, we conducted sequencing analysis using a highly conserved N-terminal shell region. The analysis showed 85.8%–100.0% nucleotide sequence identity between the strains of each combination (Additional file 4). For seven of 49 combinations, the nucleotide sequences showed 100% identity. The outbreaks of eight combinations occurred in the same season, and of these, the nucleotide sequences of three combinations showed 100% identity. We further analyzed the sequences of the partial RdRp and VP1 regions for the strains of 12 combinations, which showed 100% identity or occurred in the same season. Table 6 compares the strains in each combination and lists the epidemiological information for each outbreak.

**Table 6:** Comparison of norovirus strains in each combination and epidemiological information for each outbreak

Facility name	Outbreak strain name	Interval (month)	VP1			RdRp region	Epidemiological information	
			Genotype (variant)	Amino acid identities (%)	Domain^a^ with amino acid differences (Number of difference)	P–type	Predominant group of infection	Scale (patients/ enrollment)
PS3-5	y15-V1226-3	1^b^	GI.3	100	No difference	GI.P3	6th grade, 3rd and 4th grade	30/696
	y15-V1256-1						1st grade	13/785
PS4-2	y14-V1098-1	7^b^	GI.3	87.2	Shell (8), Protruding (62)	GI.P3	5th grade, 6th and 2nd grade	18/134
	y15-V1215-1						No data	No data
KN2-2	y16-V1450-1	0^b^	GII.2	99.8	Shell (1)	GII.P16	4-year-old class	7/60
	y16-V1509-2						0-year-old class	11/74
NH2-3	y12-V863-1	0^b^	GII.4 (Sydney 2012)	100	No difference	GII.P31	Residents on the 1st floor	7/37
	y12-V881-1						Residents on the 2nd floor	11/37
NHo5-1	y11-V657-2	0^b^	GII.4 (Den Haag 2006b)	98.9	Protruding (6)	GII.P4	Residents on the 2nd floor	28/214
	y11-V689-2						Residents on the 3rd floor	43/214
PS3-1	y16-V1355-2	0^b^	GII.2	100	No difference	GII.P16	4th grade, 1st, 5th and 6th grade	87/1028
	y16-V1387-1						1st grade, 5th and 6th grade	17/1028
PS4-7	y10-V426-3	1^b^	GII.3	99.8	Protruding (1)	GII.P12	No data	No data
	y10-V486-1						No data	No data
PS4-2	y13-V1056-4	2^b^	GII.6	100	No difference	GII.P7	Whole school	71/601
	y14-V1086-3						1st to 5th grade	55/563
KNo5-1	y12-V797-1	24	GII.4 (Sydney 2012)	99.4	Protruding (3)	GII.P31	0 to 5-year-old	18/82
	y14-V1131-5						0 to 5-year-old	15/66
NH3-2	y12-V840-1	13	GII.4 (Sydney 2012)	99.4	Shell (1), Protruding (2)	GII.P31	Whole facility	8/160
	y14-V1129-1						Whole facility	5/130
NH3-3	y12-V902-8	22	GII.4 (Sydney 2012)	99.8	Protruding (1)	GII.P31	Dementia unit	14/50
	y14-V1145-1						Dementia unit	21/50
PS3-2	y07-V195-2	48	GII.14	99.3	Protruding (4)	GII.P7	No data	No data
	y11-V717-4						1st grade	60/758

^a^Domain of GI or GII genotype was defined based on GI.1 (M87661) or GII.4 (X86557) by alignment of the VP1 amino acid sequences

^b^Outbreak occurred in the same season

**Fig. 3:**
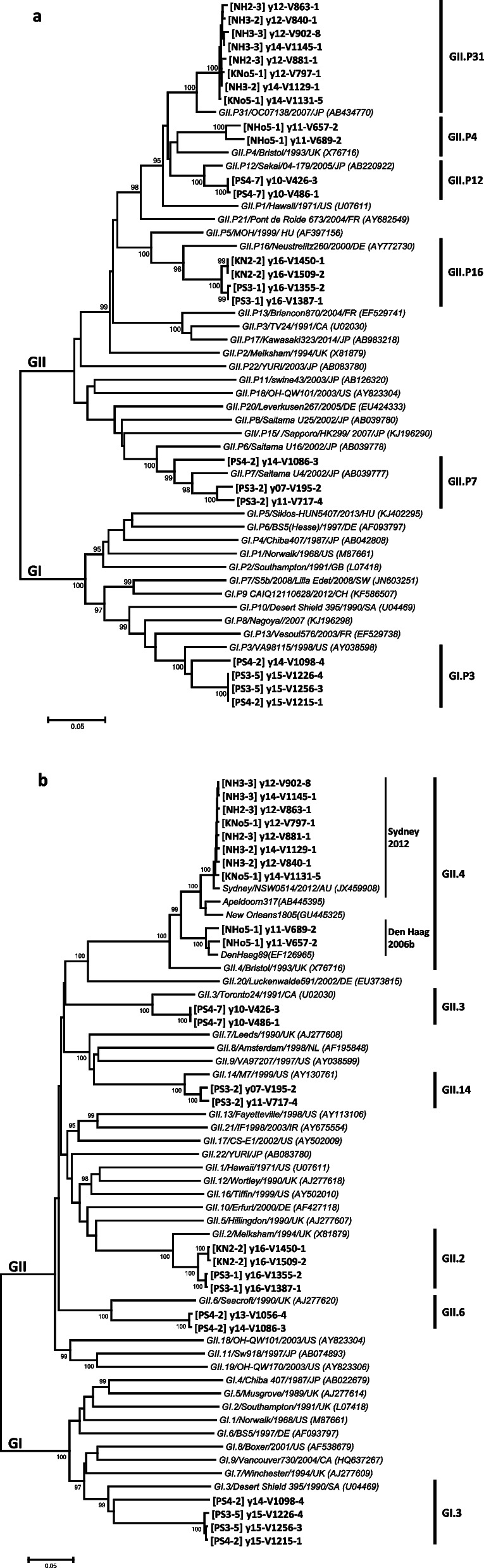
Phylogenetic analysis of norovirus. Phylogenetic trees based on **a** the partial RdRp and **b** the VP1 nucleotide sequences. The tree was constructed with the neighbor-joining method using MEGA 6 software (http://www.megasoftware.net/) with 1,000 bootstrap replicates. The percentage of bootstrap support is indicated at each node (values < 95% are omitted). The scale bar represents the number of substitutions per site. Norovirus strains for which the genes were determined in this study are denoted in bold typeface. The y13-V1056-4 strain of the partial RdRp region was omitted because the determined length was too short to construct a phylogenetic tree

Figure 3 shows the results of the phylogenetic analysis based on the nucleotide sequences of the partial RdRp and VP1 regions. P-types, defined as genotypes of the RdRp region, were consistent between outbreaks of each combination, and the phylogenetic tree showed 92.7%–100.0% nucleotide sequence identity between strains of each combination (Fig. 3a). The VP1 region showed 77.3%–99.9% nucleotide sequence identity and 87.2–100.0% deduced amino acid sequence identity between strains of each combination. Notably, the phylogenetic tree showed that the y14-V1098-4 of GI.3 was separated from y15-V1215-1 and other GI.3 strains (Fig. 3b). y14-V1098-4 and y15-V1215-1 had 12.8% amino acid differences and were different variants of GI.3, which was determined by a 5% cutoff for amino acid differences [34]. Additionally, when the shell and protruding domain of the GI or GII genotypes were defined based on the GI.1 strain (M87661) or the GII.4 strain (X86557) by temporary alignment of the VP1 amino acid sequences, most amino acid differences in each combination were in the protruding domain (Table 6). Notably, most antigenic differences in GII.4 variants map specifically on five major antigenic sites (A, C, D, E, and G) located on the P2 sub-domain in the protruding domain [35, 36]. Amino acid differences in five major antigenic sites between the strains in each combination related to GII.4 were observed in three of the five combinations.

Most outbreaks due to the same genotype in the same season occurred within 2 months. The epidemiological information suggests that these were not original infections, but infections that spread to distinct groups (Table 6).

## Discussion

Viruses associated with diarrhea are reported worldwide annually and are public health problems. Among these viruses, noroviruses are the most common causes of gastrointestinal disease outbreaks [1]. In our study, norovirus was consistently predominant in all settings throughout 10 seasons, whereas sapovirus, RVA, and RVC did not exceed 10% in any season. The distribution of norovirus genotypes in K/Ns, PSs, and NHs was different, similar to our previous study [3]. The infants attending K/Ns have immature immunity and are in closer contact with adults than school children, therefore they may also be affected by the dominant genotype in adults among various genotypes. School children have a wider sphere of activity than infants, come in contact with more various genotypes. Conversely, transmission in NHs mainly occurs through person-to-person transmission through helpers or visitors. It is consider that the genotype detected in NHs was homogeneous because they have limited mobility and live in a confined space. Thus, the distribution of genotype is likely dependent upon the facility, especially the patient age [3].

To consider herd immunity at the facility level, we clarified the status of repeated AG outbreaks at the same facilities in Yokohama, Japan. When considering herd immunity at the facility level, the influence of different population, such as transition of people in facilities over time, cannot be avoided. However, in this study, we conducted analyses without considering these influences because such factors cannot be fully captured in these facilities. PSs and K/Ns had larger proportions of repeated outbreaks than other settings. Thus, younger people such as infants and children may be more susceptible to various viral genotypes because they have not acquired sufficient immunity [3].

Among 227 facilities that reported repeated AG outbreaks, 173 (76.2%) had only norovirus outbreaks. Of these, different genotype combinations occurred more frequently than the same genotype combinations, which is consistent with other reports in which most repeat infections were due to a different genotype than those of previous infections [10, 11]. Additionally, for facilities that experienced two norovirus outbreaks, the average interval between outbreaks was longer for groups with the same combinations than for groups with different genogroup or genotype combinations. Our results support those of a previous report that found norovirus genotype-specific herd immunity may influence norovirus outbreaks at the facility level [12]. Moreover, the average interval between norovirus combination outbreaks was longer than that of other virus combinations, possibly because cross-reactivity between allogeneic viruses is higher than that between heterologous viruses.

Multiple AG outbreaks occurred during the same season at the same facilities and most of these facilities had different norovirus genotypes or virus combinations, suggesting that herd immunity was maintained at the facility level for at least the same season. An early challenge study conducted on volunteers demonstrated poor cross-reactivity among viruses from different norovirus genogroups [10]. Recent reports showed that repeat infections with the same genotype were rare for a certain time period, possibly because of the immune protection obtained from initial infection of the same genotype [11, 14, 37]. Our results were consistent with these findings.

Additionally, analysis of genetic relationships and natural history patterns identified groupings of certain genotypes into larger related clusters designated as immunotypes [34]. This study showed that most reinfections occurred with viruses of different immunotypes. Our results support this conclusion and show that all outbreaks due to different genotypes that occurred during the same season at the same facility were all caused by different immunotypes. Our epidemiological information suggested that most outbreaks due to the same norovirus genotypes in the same season may have been caused by the spread of infection to distinct groups. However, in kindergartens, nursery schools, and primary schools in Japan, children typically enroll every April; thus, new groups of children with different susceptibilities may have partially influenced these results.

During the 10-year study period, the same facilities experienced outbreaks of the same norovirus genotype combination by GII.4, GII.2, GII.6, GII.3, GII.14, and/or GI.3. These genotypes have been dominant in Yokohama and have been detected every season, although the frequency of detection fluctuates [3, 26]. The average interval between these outbreaks was 31.2 months during the study period. The average intervals between outbreaks differed by genotype and were 39.9 months for non-GII.4 genotypes cases and 18.6 months for GII.4 cases, and significantly differed between groups (*P* < 0.05). The average intervals were shorter for GI.3 and GII.4 than for other genotype combinations. For two combinations of GI.3, the intervals may have been much shorter because one was an expanded infection to distinct age groups, and the other was a rare case due to a different variant type. Although previous studies assumed that individual genotypes represent strains with similar phenotypes, recent studies have shown that evolution in some genotypes, such as GII.4, is sufficient to generate mutant clusters with new ligand-binding characteristics and antigenic properties [38, 39]. Because y14-V1098-4 and y15-V1215-1 had 12.8% amino acid differences in VP1 and the differences concentrated in the protruding domain, where there are predicted antigenic sites, the strain of the second outbreak (y15-V1215-1) in PS4-2 may have had different phenotypes from the strain of the first outbreak (y14-V1098-4).

Non-GII.4 genotypes sustain a low number of intragenotypic variants with a limited number of amino acids differences, even if they occur decades apart, whereas GII.4 produces the most intragenotypic variants [34]. Antigenicity changes in GII.4 were reported to be associated with amino acid substitutions in the protruding domains of VP1 proteins; additionally, even within each GII.4 variant, amino acid changes occurred in VP1, despite the presence of evolutionary constraints [26, 40]. VP1 sequence analysis of some GII.4 strains in this study revealed that most amino acid substitutions were detected in the protruding domain. Notably, there were amino acid differences in some combinations related to GII.4 in five major antigenic sites located on the P2 sub-domain. These antigenicity changes may have facilitated escape from herd immunity, leading to successive outbreaks in the short term with GII.4 variants. Additionally, an age-related decline in immune function is partially responsible for the increased prevalence of infectious diseases [41]. Most outbreaks that occurred in NHs were GII.4, which might have contributed to the shorter average interval between outbreaks.

Sakon et al. reported that genotype-specific herd immunity in infants and young children lasts for at least a few years, thereby influencing the endemic norovirus genotype in the next season [12]. The average intervals between outbreaks were 36.4 months for K/Ns and 35.7 months for PSs, suggesting that genotype-specific herd immunity lasts several years, which is consistent with the findings of Sakon et al. Additionally, our data indicated that herd immunity may influence endemic norovirus genotypes in the next season, especially in PSs (Fig. 1).

Recombination frequently occurs in the open reading frame (ORF)1/ORF2 overlap and is associated with antigenic shift [42]. Although we conducted sequence analyses of the RdRp region for the 12 strain combinations to consider the possibility of recombination, the P-type of each combination was consistent.

RVA vaccination provides protection against severe RVA. It does not confer sterilizing immunity, but may have indirect protective effects for unimmunized individuals as a result of others being immunized [43]. Antibodies to norovirus may protect against certain genotypes of norovirus infection, and vaccination is a way to actively acquire antibodies. Norovirus vaccinations may alleviate public health problems similar to RVA vaccinations. However, norovirus vaccine development has many difficulties and limitations, partly because of the limited availability of norovirus cell cultures, complexity of protective immunity against norovirus, antigenic variation among and within genogroups and genotypes, and unknown effects of pre-exposure history [44, 45]. We believe that our study is informative for public health, but is limited geographically to Yokohama, Japan. Comprehensive studies of facility-level herd immunity remain scarce. Norovirus genotype trends differ depending on year, area, and age group, and steady global surveillance and further studies are needed to influence future vaccine policy decisions.

## Conclusions

We determined the statuses of repeated AG outbreaks at the same facilities in Yokohama, Japan. Most of these facilities experienced a combination of noroviruses. Our data indicate that herd immunity at the facility level was maintained for at least the same season. Norovirus genotype-specific herd immunity was maintained for an average of 31.2 months at the facility level during the study period, and the intervals differed depending on genotype. However, we conducted our analyses without considering the influence of different populations within each facility, so the results should be interpreted with caution.

## Supplementary Information

**Additional file 1.** Primers for amplification and sequencing of the partial RdRp and VP1 regions of norovirus strains.

**Additional file 2.** Distribution of settings where AG viruses were detected in Yokohama, Japan, 2007–2017.

**Additional file 3. **Details regarding multiple outbreaks in facilities.

**Additional file 4.** Norovirus outbreaks due to the same genotype in the same facility.

## Data Availability

The datasets generated and analyzed during the current study are available in the DDBJ/GenBank/EMBL databases under accession numbers LC720153–LC720176. https://www.ncbi.nlm.nih.gov/genbank/.

## Abbreviations

AG: Acute gastroenteritis
K/Ns: Kindergarten/nursery schools
NHs: Nursing homes for older adults
PSs: Primary schools
RdRp: RNA-dependent RNA polymerase
RVA: Rotavirus A
RVC: Rotavirus C

## References

[1] Green KY, Knipe DM, Howley P (2013). Caliciviridae: the noroviruses. Field’s Virology.

[2] Oka T, Wang Q, Katayama K, Saif LJ (2015). Comprehensive review of human sapoviruses. Clin Microbiol Rev.

[3] Kumazaki M, Usuku S (2016). Norovirus genotype distribution in outbreaks of acute gastroenteritis among children and older people: an 8-year study. BMC Infect Dis.

[4] Kumazaki M, Usuku S (2014). Epidemiological and genetic analysis of human group C rotaviruses isolated from outbreaks of acute gastroenteritis in Yokohama, Japan, between 2006 and 2012. Arch Virol.

[5] Usuku S, Kumazaki M (2014). A gastroenteritis outbreak attributed to sapovirus genogroup v in Yokohama. Japan Jpn J Infect Dis.

[6] Kroneman A, Vega E, Vennema H, Vinjé J, White PA, Hansman G (2013). Proposal for a unified norovirus nomenclature and genotyping. Arch Virol.

[7] Vinjé J (2015). Advances in laboratory methods for detection and typing of norovirus. J Clin Microbiol.

[8] Chhabra P, de Graaf M, Parra GI, Chan MC, Green K, Martella V (2019). Updated classification of norovirus genogroups and genotypes. J Gen Virol.

[9] Simmons K, Gambhir M, Leon J, Lopman B (2013). Duration of immunity to norovirus gastroenteritis. Emerg Infect Dis.

[10] Wyatt RG, Dolin R, Blacklow NR, DuPont HL, Buscho RF, Thornhill TS (1974). Comparison of three agents of acute infectious nonbacterial gastroenteritis by cross-challenge in volunteers. J Infect Dis.

[11] Saito M, Goel-Apaza S, Espetia S, Velasquez D, Cabrera L, Loli S (2014). Multiple norovirus infections in a birth cohort in a Peruvian Periurban community. Clin Infect Dis.

[12] Sakon N, Yamazaki K, Nakata K, Kanbayashi D, Yoda T, Mantani M (2015). Impact of genotype-specific herd immunity on the circulatory dynamism of norovirus: a 10-year longitudinal study of viral acute gastroenteritis. J Infect Dis.

[13] Harada S, Oka T, Tokuoka E, Kiyota N, Nishimura K, Shimada Y (2012). A confirmation of sapovirus re-infection gastroenteritis cases with different genogroups and genetic shifts in the evolving sapovirus genotypes, 2002–2011. Arch Virol.

[14] Sánchez GJ, Mayta H, Pajuelo MJ, Neira K, Xiaofang L, Cabrera L (2018). Epidemiology of Sapovirus Infections in a Birth Cohort in Peru. Clin Infect Dis.

[15] Matthijnssens J, Ciarlet M, McDonald SM, Attoui H, Bányai K, Brister JR (2011). Uniformity of rotavirus strain nomenclature proposed by the Rotavirus Classification Working Group (RCWG). Arch Virol.

[16] Velázquez FR, Matson DO, Calva JJ, Guerrero L, Morrow AL, Carter-Campbell S (1996). Rotavirus infection in infants as protection against subsequent infections. N Engl J Med.

[17] Fine P, Eames K, Heymann DL (2011). "Herd immunity": a rough guide. Clin Infect Dis.

[18] Hoehne M, Schreier E (2006). Detection of Norovirus genogroup I and II by multiplex real-time RT- PCR using a 3'-minor groove binder-DNA probe. BMC Infect Dis.

[19] Jothikumar N, Lowther JA, Henshilwood K, Lees DN, Hill VR, Vinjé J (2005). Rapid and sensitive detection of noroviruses by using TaqMan-based one-step reverse transcription-PCR assays and application to naturally contaminated shellfish samples. Appl Environ Microbiol.

[20] Oka T, Katayama K, Hansman GS, Kageyama T, Ogawa S, Wu FT (2006). Detection of human sapovirus by real-time reverse transcription-polymerase chain reaction. J Med Virol.

[21] Freeman MM, Kerin T, Hull J, McCaustland K (2008). Gentsch J Enhancement of detection and quantification of rotavirus in stool using a modified real-time RT-PCR assay. J Med Virol.

[22] Logan C, O'Leary JJ, O'Sullivan N (2006). Real-time reverse transcription-PCR for detection of rotavirus and adenovirus as causative agents of acute viral gastroenteritis in children. J Clin Microbiol.

[23] Kageyama T, Shinohara M, Uchida K, Fukushi S, Hoshino FB, Kojima S (2004). Coexistence of multiple genotypes, including newly identified genotypes, in outbreaks of gastroenteritis due to Norovirus in Japan. J Clin Microbiol.

[24] Kojima S, Kageyama T, Fukushi S, Hoshino FB, Shinohara M, Uchida K (2002). Genogroup-specific PCR primers for detection of Norwalk-like viruses. J Virol Methods.

[25] Saito H, Saito S, Kamada K, Harata S, Sato H, Morita M (1998). Application of RT-PCR designed from the sequence of the local SRSV strain to the screening in viral gastroenteritis outbreaks. Microbiol Immunol.

[26] Kumazaki M, Usuku S (2015). Genetic analysis of norovirus GII.4 variant strains detected in outbreaks of gastroenteritis in Yokohama, Japan, from the 2006–2007 to the 2013–2014 seasons. PLoS One..

[27] Nayak MK, Balasubramanian G, Sahoo GC, Bhattacharya R, Vinje J, Kobayashi N (2008). Detection of a novel intergenogroup recombinant Norovirus from Kolkata. India Virology.

[28] Vennema H, de Bruin E (2002). Koopmans M Rational optimization of generic primers used for Norwalk-like virus detection by reverse transcriptase polymerase chain reaction. J Clin Virol.

[29] Jennifer L. Cannon, Leslie Barclay, Nikail R. Collins, Mary E. Wikswo, Christina J. Castro, et al. Genetic and epidemiologic trends of norovirus outbreaks in the United States from 2013 to 2016 demonstrated emergence of novel GII.4 recombinant viruses. J Clin Microbiol. 2017; 55: 2208–2221. 10.1128/JCM.00455-17 PMC5483924 28490488

[30] Iritani N, Vennema H, Siebenga JJ, Siezen RJ, Renckens B, Seto Y, et al. Genetic analysis of the capsid gene of genotype GII.2 noroviruses. J Virol. 2008; 82: 7336–7345. 10.1128/JVI.02371-07 PMC2493331 18480447

[31] Boon D, Mahar JE, Abente EJ, Kirkwood CD, Purcell RH, Kapikian AZ, et al. Comparative evolution of GII.3 and GII.4 norovirus over a 31-year period. J Virol. 2011; 85: 8656–8666. 10.1128/JVI.00472-11 PMC3165818 21715504

[32] Okada M, Ogawa T, Kaiho I, Shinozaki K (2005). Genetic analysis of noroviruses in Chiba Prefecture, Japan, between 1999 and 2004. J Clin Microbiol.

[33] Kroneman A, Vennema H, Deforche K, v d Avoort H, Peñaranda S, Oberste MS, et al. An automated genotyping tool for enteroviruses and noroviruses. J Clin Virol. 2011; 51: 121–125. 10.1016/j.jcv.2011.03.006 21514213

[34] Parra GI, Squires RB, Karangwa CK, Johnson JA, Lepore CJ, Sosnovtsev SV (2017). Static and evolving norovirus genotypes: implications for epidemiology and immunity. PLoS Pathog.

[35] Lindesmith LC, Costantini V, Swanstrom J, Debbink K, Donaldson EF, Vinjé J, et al. Emergence of a norovirus GII.4 strain correlates with changes in evolving blockade epitopes. J Virol. 2013; 87: 2803–2813. 10.1128/JVI.03106-12 PMC3571402 23269783

[36] Tohma K, Lepore CJ, Gao Y, Ford-Siltz LA, Parra GI. Population genomics of GII.4 noroviruses reveal complex diversification and new antigenic sites involved in the emergence of pandemic strains. mBio. 2019; 10(5): e02202–19. 10.1128/mBio.02202-19 PMC6759766 31551337

[37] Ayukekbong JA, Fobisong C, Tah F, Lindh M, Nkuo-Akenji T, Bergström T (2014). Pattern of circulation of norovirus GII strains during natural infection. J Clin Microbiol.

[38] Donaldson EF, Lindesmith LC, Lobue AD, Baric RS (2010). Viral shape-shifting: norovirus evasion of the human immune system. Nat Rev Microbiol.

[39] Lindesmith LC, Donaldson EF, Lobue AD, Cannon JL, Zheng DP, Vinje J, et al. Mechanisms of GII.4 norovirus persistence in human populations. PLoS Med. 2008; 5(2): e31. 10.1371/journal.pmed.0050031 PMC2235898 18271619

[40] Sato H, Yokoyama M, Nakamura H, Oka T, Katayama K, Takeda N (2017). Evolutionary constraints on the norovirus pandemic variant GII.4_2006b over the five-year persistence in Japan. Front Microbiol.

[41] Weiskopf D, Weinberger B, Grubeck-Loebenstein B (2009). The aging of the immune system. Transpl Int.

[42] Bull RA, Hansman GS, Clancy LE, Tanaka MM, Rawlinson WD, White PA (2005). Norovirus recombination in ORF1/ORF2 overlap. Emerg Infect Dis.

[43] Payne DC, Staat MA, Edwards KM, Szilagyi PG, Weinberg GA, Hall CB (2011). Direct and indirect effects of rotavirus vaccination upon childhood hospitalizations in 3 US Counties, 2006–2009. Clin Infect Dis.

[44] Riddle MS, Walker RI (2016). Status of vaccine research and development for norovirus. Vaccine.

[45] Ford-Siltz LA, Tohma K, Parra GI (2021). Understanding the relationship between norovirus diversity and immunity Gut Microbes. Gut Microbes.

