# Supplementary material for: Influence of herd immunity on norovirus: a long-term field study of repeated viral gastroenteritis outbreaks at the same facilities
Source: BMC Infect Dis. 2023 Apr 26;23:265. doi: 10.1186/s12879-023-08251-7 (PMC10132420; doi:10.1186/s12879-023-08251-7)
Supplement: Supplementary file 2 — Additional file 2. Distribution of settings where AG viruses were detected in Yokohama, Japan, 2007–2017. [file 12879_2023_8251_MOESM2_ESM.pptx]

## Slide 1
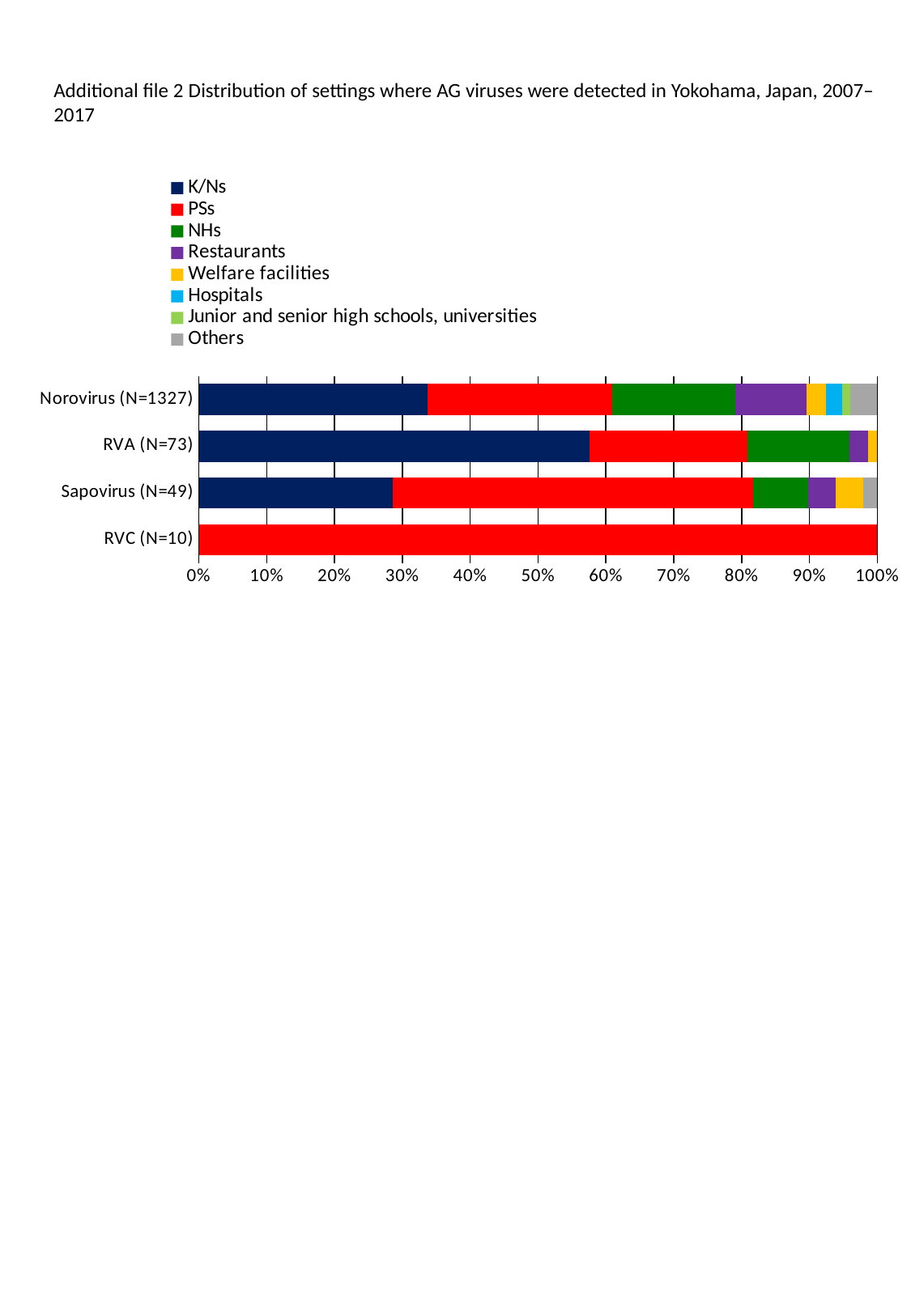

Additional file 2 Distribution of settings where AG viruses were detected in Yokohama, Japan, 2007–2017
### Chart
| Category | K/Ns | PSs | NHs | Restaurants | Welfare facilities | Hospitals | Junior and senior high schools, universities | Others |
|---|---|---|---|---|---|---|---|---|
| RVC (N=10) | None | 10.0 | None | None | None | None | None | None |
| Sapovirus (N=49) | 14.0 | 26.0 | 4.0 | 2.0 | 2.0 | None | None | 1.0 |
| RVA (N=73) | 42.0 | 17.0 | 11.0 | 2.0 | 1.0 | None | None | None |
| Norovirus (N=1327) | 448.0 | 360.0 | 241.0 | 139.0 | 39.0 | 31.0 | 16.0 | 53.0 |
